# Combination of Parenteral Amino Acid Infusion and Intermittent Loading Exercise Ameliorates Progression of Postoperative Sarcopenia in Rat Model

**DOI:** 10.3390/nu16081218

**Published:** 2024-04-19

**Authors:** Akira Wada, Hayato Yamashita, Ayaka Togashi, Shunsuke Ogawa, Arashi Muroi, Satoshi Kido, Shigeki Furuya

**Affiliations:** 1 Naruto Research Institute, Research and Development Center, Otsuka Pharmaceutical Factory, Inc., Naruto 772-8601, Japan; yamashita.hayato@otsuka.jp (H.Y.); ogawa.shunsuke@otsuka.jp (S.O.); muroi.arashi@otsuka.jp (A.M.); kido.satoshi@otsuka.jp (S.K.); 2 Department of Innovative Science and Technology for Bio-Industry, Graduate School of Bioresource and Bioenvironmental Sciences, Kyushu University, Fukuoka 819-0395, Japan; 3 Innovative Bio-Architecture Center, Faculty of Agriculture, Kyushu University, Fukuoka 819-0395, Japan; 4 Environmental Control Center for Experimental Biology, Kyushu University, Fukuoka 819-0395, Japan

**Keywords:** amino acid, parenteral nutrition, peripheral parenteral nutrition, sarcopenia, skeletal muscle, muscle atrophy, postoperative nutritional management, exercise

## Abstract

Postoperative sarcopenia is associated with poor outcomes in hospitalized patients. However, few studies have focused on short-term postoperative sarcopenia. Furthermore, the influence of nutritional management using amino acids (AAs) comprising a peripheral parenteral nutrition (PPN) solution and its combination with exercise (Exc) is unclear. Hence, we established a postoperative sarcopenic rat model to evaluate the effects of parenteral AA infusion combined with Exc on skeletal muscles and investigate the underlying mechanisms involved in the amelioration of muscle atrophy. Male F344 rats underwent surgery followed by hindlimb suspension (HS) for 5 days. The rats were divided into AA (−), AA (+), AA (−)-Exc, and AA (+)-Exc groups. They were continuously administered a PPN solution with or without AA at 98 kcal/kg/day. The Exc groups were subjected to intermittent loading for 1 h per day. Postoperative sarcopenic rats exhibited decreased muscle strength and mass and an upregulated ubiquitin–proteasome system, autophagy–lysosome system, and fast-twitch fiber-related genes, especially in the AA (−) group. The AA (+)-Exc group exhibited attenuated decreased muscle strength, increased gastrocnemius mass, and a suppressed upregulation of muscle atrophy- and fast-twitch fiber-related genes. Therefore, parenteral AA infusion combined with Exc may be effective in preventing postoperative sarcopenia in hospitalized patients.

## 1. Introduction

Sarcopenia is a muscle disease that accelerates the loss of muscle mass and function [1]. It is classified as primary (age-related) and secondary sarcopenia. Secondary sarcopenia is associated with disease, inactivity, and malnutrition [2]. In particular, surgical patients experience conditions such as disease, surgery-related inflammation, bed rest after surgery, and malnutrition or inadequate nutritional intake [3]. The prevalence of sarcopenia in hospitalized or abdominal surgery patients is reported to range from 21.1% to 34.5% [4,5,6,7,8]. Sarcopenia is associated with adverse outcomes, including hospital admission; worsened walking ability in acutely ill older adults [6]; postoperative complications; and the hospital mortality of patients undergoing abdominal surgery [7], gastrectomy [8], and acute care and emergency surgery [9]. Additionally, sarcopenia is linked to higher hospital and long-term mortality in emergency abdominal surgery patients [10]. Furthermore, it has been reported that approximately 12–15% of elderly patients without sarcopenia at hospital admission develop sarcopenia during hospitalization [11,12]. Moreover, skeletal muscle loss within a week of esophagectomy is associated with worse overall survival [13]. Therefore, the prevention of sarcopenia is critical for hospitalized elderly patients.

It is well documented that skeletal muscle is maintained by the balance between muscle protein degradation (MPD) and synthesis (MPS). In MPD, the ubiquitin–proteasome and autophagy–lysosome systems play roles in the major proteolysis pathway [14]. The expression of F-box protein (MAFbx)/Atrogin-1 and muscle-specific RING finger 1 (MuRF1) is increased in various muscle atrophy conditions involved in the ubiquitin–proteasome system [15]. Autophagy, accompanied by mitochondrial dysfunction, also contributes to muscle atrophy. Muscle disuse activates autophagy-related gene expression [16]. Forkhead box protein O (FOXO) transcription factors regulate these two protein degradation pathways [17]. In MPS, the mechanistic target of the rapamycin complex 1 (mTORC1)-mediated phosphorylation of its downstream factor p70 ribosomal protein S6 kinase (p70S6K) and eukaryotic translation initiation factor 4E-binding protein 1 (4E-BP1) promotes protein synthesis [18]. Furthermore, muscle atrophy-related changes in muscle fibers and slow-to-fast twitch transitions are associated with muscle disuse, such as bed rest and hindlimb suspension (HS) [19].

There are several reports on the effect of physical and/or nutritional interventions for sarcopenia, particularly in older and surgical patients subjected to dietary protein or amino acid (AA) supplementation, exercise (Exc), or rehabilitation [20,21]. Oral nutrition support intended to increase protein content with rehabilitation or enhanced physical activity significantly enhanced grip strength in hospitalized older adults. However, muscle mass was not evaluated in the meta-analysis because of the inconsistency in assessment in the selected studies [20]. Another systematic review and meta-analysis indicated that exercise interventions, but not nutritional interventions, significantly improved muscle mass and strength in the surgical population [21]. Furthermore, a recent randomized controlled trial in patients with colorectal cancer found that postoperative Exc also increased muscle mass and shortened the length of hospital stay [22].

Parenteral nutrition (PN) is usually used for nutritional management in patients who cannot use the oral/enteral route, those with insufficient energy intake, or as a bridge to enteral nutrition (EN). Particularly in cases with a short postoperative period, a peripheral parenteral nutrition (PPN) solution, which is generally designed to have lower energy and sufficient amino acid content, is usually used via the peripheral route [23,24]. The postoperative administration of PPN with an oral nutritional supplement (ONS) prevented body weight loss and skeletal muscle loss compared with 4.3% glucose fluid in patients with gastrectomy [25]. However, to the best of our knowledge, the influence of PPN, especially AA infusion, has not been explored. In addition, the effect of PPN combined with Exc on sarcopenia in patients with a short postoperative period remains unclear.

Therefore, in this study, we established a postoperative sarcopenic rat model and evaluated the effects of parenteral AA infusion combined with Exc on muscle strength and mass, including the underlying mechanisms associated with muscle atrophy.

## 2. Materials and Methods

### 2.1. Animals

Male F344/NSlc rats (age, 11 weeks; body weight, 190–220 g) were purchased from Japan SLC, Inc. (Shizuoka, Japan). All rats were housed under constant temperature (23 ± 3 °C) and humidity (55 ± 15%) conditions with a 12 h/12 h light/dark cycle. They were provided free access to the AIN-93G diet [26] purchased from Oriental Yeast Co., Ltd. (Tokyo, Japan) and water during the acclimatization period (7 days). This study was approved by the Committee for the Care and Use of Laboratory Animals of Otsuka Pharmaceutical Factory, Inc., Japan (approval number: OPFCAE-21-010; approval date: 12 January 2021).

### 2.2. Experimental Design and Operative Procedures

#### 2.2.1. Models of Postoperative Sarcopenia and Intermittent Loading

All rats were fasted overnight the day before surgery (day −1). They were randomly divided into five groups based on their body weight on day 0 (206.8 ± 12.3 g) and hindlimb muscle strength on day −1 (110.4 ± 9.8 m·Nm): normal, no AA infusion (AA (−)), with AA infusion (AA (+)), no AA infusion with Exc (AA (−)-Exc), and AA infusion with Exc (AA (+)-Exc) (n = 10/group). A catheter was inserted into the jugular vein of the rats when they were sedated using a combination of three anesthetic agents (medetomidine, midazolam, and butorphanol). As postoperative sarcopenic models, each group of rats, except for the normal group, underwent surgery followed by catheter placement and was subjected to HS for 5 days. Laparotomy was performed, and the intestine was rubbed with medical gauze for 3 min and exposed for 20 min, as previously described [27]. Following surgery, the rats were placed inside individual cages and suspended by their tails using adhesive tape and wearing equipment. HS was performed with their tail angled at approximately 30° head-down, and the rats were allowed to move freely in their cages using their forelimbs, as described in a previous report [28]. Rats in the AA (−)-Exc and AA (+)-Exc groups were released from HS for 1 h/d for the intermittent loading of Exc in accordance with previous studies [29,30]. During this period, the rats were allowed to move freely using their forelimbs and hindlimbs.

#### 2.2.2. Nutritional Management

Rats in the normal group were fed an AIN-93G diet ad libitum and infused continuously with saline (isotonic sodium chloride solution) via the catheter. The other groups were infused with commercially available AA (−) or AA (+) experimental solutions, which are used in clinical practice. The AA (−) solution (Physio 35; Otsuka Pharmaceutical Factory, Inc., Tokushima, Japan) consisted of 10% glucose and electrolytes. The AA (+) solution (BFLUID: Otsuka Pharmaceutical Factory, Inc., Tokushima, Japan) consisted of 7.5% glucose, 3% AAs, and electrolytes. The daily energy dosage was 98 kcal/kg/day. This energy dosage for rats corresponds to 840 kcal/60 kg in humans for the clinical setting of 2000 mL of the AA (+) PN solution administered via the peripheral vein. This is based on the basal metabolic rate of rats, which is approximately seven times higher than that of humans [31]. The AA (−) and AA (+) solutions were administered continuously for 5 days. Table 1 lists the compositions of the experimental solutions and daily doses. A rat in the AA (−)-Exc group died, and a rat in the AA (−) group was excluded because of suspicion of infection during the experimental period.

#### 2.2.3. Tissue and Blood Sampling

Five days after the administration of the test solutions, the rats were euthanized using isoflurane anesthesia. Blood was collected through the caudal vena cava, and plasma was obtained by centrifugation. The gastrocnemius, soleus, extensor digitorum longus (EDL), plantaris, and tibialis anterior (TA) muscles of the left hindlimb were excised, weighed, and snap-frozen in liquid nitrogen. Plasma and hindlimb muscle samples were stored at −80 °C until analysis. The entire right hindlimb was excised and fixed in 10% (*v*/*v*) neutral buffered formalin for further histological analysis.

### 2.3. Measurements

#### 2.3.1. Body Weight and Composition

Body weight and lean body mass were measured prior to surgery (day 0) and euthanasia (day 5), respectively. Lean body mass was measured using an Echo MRI device (Echo Medical Systems LLC, Houston, TX, USA).

#### 2.3.2. Muscle Weight and Gastrocnemius Myofiber Cross-Sectional Area

The weights of the left hindlimb gastrocnemius, soleus, EDL, plantaris, and TA muscles were measured immediately after excision. The formalin-fixed right-side hindlimb was embedded in paraffin, sectioned into 4 μm thick slices, and stained with Sirius red using 0.1% Sirius red in a picric acid solution for 1 h. The stained sections were observed under a light microscope (BX-53, Olympus Corp., Tokyo, Japan), and one view of almost the same region in each cross-sectional area (CSA) of the gastrocnemius myofibers was measured using image analysis software (WinROOF2018, Mitani Corporation, Fukui, Japan).

#### 2.3.3. Hindlimb Muscle Strength

To measure hindlimb muscle strength, the maximum isometric contraction force of the right hindlimb (the ankle joint angle was positioned at 90°) was measured on the day before surgery (day −1) and on the day of euthanasia (day 5). The maximal isometric contraction force was measured using an animal torque measurement device (T.K.K.5813; Takei Scientific Instruments Co., Ltd., Niigata, Japan) with the electrical stimulation (30 V, 100 Hz, 1 s) of the sciatic nerve under 2.0% isoflurane anesthesia.

#### 2.3.4. Quantitative Real-Time Polymerase Chain Reaction

Snap-frozen whole gastrocnemius muscle samples were powdered using a multi-bead shocker (Yasui Kikai Corp., Osaka, Japan). Total RNA from the gastrocnemius muscle was extracted using an RNeasy Fibrous Tissue Mini Kit (Qiagen, Hilden, Germany). cDNA was synthesized from total RNA using a PrimeScript RT Reagent Kit (Takara, Kyoto, Japan). The mRNA expression levels of MuRF-1/Trim63 (Rn00590197_m1), Atrogin1/Fbxo32 (Rn00591730_m1), Ulk1 (Rn02108711_s1), LC3b/Map1lc3b (Rn02132764_s1), p62/Sqstm1 (Rn00709977_m1), Gabarap (Rn00490680_g1), Bnip3 (Rn00821446_g1), Foxo1 (Rn01494868_m1), Foxo3 (Rn01441087_m1), Gadd45a (Rn00577049_m1), Eif4ebp1 (Rn00587824_m1), Myh7 (Rn01488777_g1), Myh2 (Rn01470656_m1), Myh1 (Rn01751056_m1), and Myh4 (Rn01496087_g1) were assessed using TaqMan Gene Expression Assays (Thermo Fisher Scientific, Waltham, MA, USA). Quantitative real-time polymerase chain reaction was performed using an ABI 7500 Fast Real-Time PCR System (Thermo Fisher Scientific Inc., Waltham, MA, USA) under the following conditions: 50 °C for 2 min, 95 °C for 2 min, and 40 cycles of 95 °C for 3 s and 60 °C for 30 s. mRNA expression was quantified using the 2^−ΔΔCt^ method, with Actb (Rn00667869_m1) as the reference gene.

#### 2.3.5. Western Blot Analysis

Powdered whole gastrocnemius muscle samples were homogenized in RIPA buffer (Cell Signaling Technology, Danvers, MA, USA) supplemented with protease and phosphatase inhibitor cocktails. Homogenates were centrifuged, and the supernatants were collected as protein extracts. The concentration of the protein extracts was measured using a Pierce BCA Protein Assay Kit (Thermo Fisher Scientific Inc., Waltham, MA, USA). Protein samples were mixed with 3× SDS Loading buffer (Cell Signaling Technology, Danvers, MA, USA) and heated at 95 °C for 5 min. Equal amounts of protein samples (40 μg) were fractionated by 12.5% SDS–polyacrylamide gel electrophoresis, transferred to a PVDF membrane, and blocked with Blocking One-P (Nacalai Tesque, Inc., Kyoto, Japan) for 30 min at room temperature. Membranes were incubated with primary antibodies (Cell Signaling Technology, Danvers, MA, USA) against 4E-BP1 (1:1000, #9452) and phospho 4E-BP1 (Ser65) (1:1000, #9451) at 4 °C overnight. Subsequently, membranes were incubated with anti-rabbit IgG and HRP-linked secondary antibodies (1:3000, #7074) for 1 h at room temperature. The bound antibodies were visualized using a chemiluminescent reagent (Luminata Western HRP substrate; EMD Millipore Corporation, Burlington, MA, USA). The chemiluminescent signals were quantified using a Western blotting and chemiluminescence imaging system (FUSION SOLO. 7S EDGE; Vilber Lourmat, Collégien, France). The phosphorylation of 4E-BP1 is represented as γ forms to the total 4E-BP1 (α + β + γ forms) protein expression level [32] and as phospho 4E-BP1 (Ser65) levels normalized to the total 4E-BP1 protein level.

#### 2.3.6. Plasma AA Analysis

Plasma samples were deproteinized with acetonitrile, the supernatants were subjected to liquid chromatography–mass spectrophotometry (LC-MS2020, Shimadzu Corporation, Kyoto, Japan), and the concentration of each AA was measured.

### 2.4. Statistical Analyses

Data are presented as means ± standard deviations (SDs). The homogeneity of variance was analyzed using Bartlett’s test. According to the results of Bartlett’s test (homoscedasticity: *p* ≥ 0.05 or heteroscedasticity: *p* < 0.05), statistical significance was assessed using one-way analysis of variance (ANOVA) or non-parametric ANOVA (Kruskal–Wallis test) followed by either the multiple comparisons test of Dunnett (parametric) or the Dunnett-type joint-ranking (non-parametric) test in comparison with the normal group. They were assessed using either Tukey’s multiple comparisons test (parametric) or the Tukey-type joint-ranking (non-parametric) test among the postoperative sarcopenic rats in the AA (−), AA (+), AA (−)-Exc, and AA (+)-Exc groups. Statistical analyses were performed using EXSUS version 10.0 software (CAC Croit Corporation, Tokyo, Japan). Statistical significance was set at a *p*-value of less than 0.05.

## 3. Results

### 3.1. Body Weight and Lean Mass

**Figure 1:**
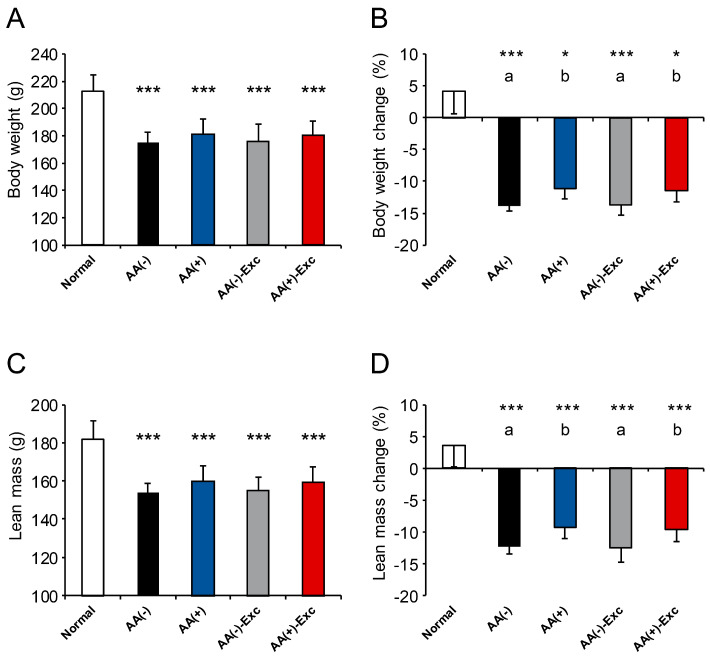
The effects of AA and Exc on body weight and lean mass. (**A**) Body weight at the end of the experiment. (**B**) Changes in body weight during the experiment. (**C**) Lean mass at the end of the experiment. (**D**) Lean mass change during the experiment. Data are presented as means ± SDs (n = 9–10 per group). * *p* < 0.05, *** *p* < 0.001, compared to the normal group using Dunnett-type multiple comparison tests among all groups. Different letters indicate significant differences among the postoperative sarcopenic rats in the AA (−), AA (+), AA (−)-Exc, and AA (+)-Exc groups using Tukey-type multiple comparison tests (*p* < 0.05). AA, amino acid; Exc, exercise; SD, standard deviation.

The final body weight, lean mass, and rates of change in body weight and lean mass were significantly lower in all postoperative sarcopenic rat model groups than in the normal group (*p* < 0.05 and *p* < 0.001, respectively). The rates of change in body weight and lean mass in the AA (+) and AA (+)-Exc groups were significantly higher than those in the AA (−) and AA (−)-Exc groups (*p* < 0.05, Figure 1).

### 3.2. Skeletal Muscle Mass and Gastrocnemius Myofiber CSA

**Figure 2:**
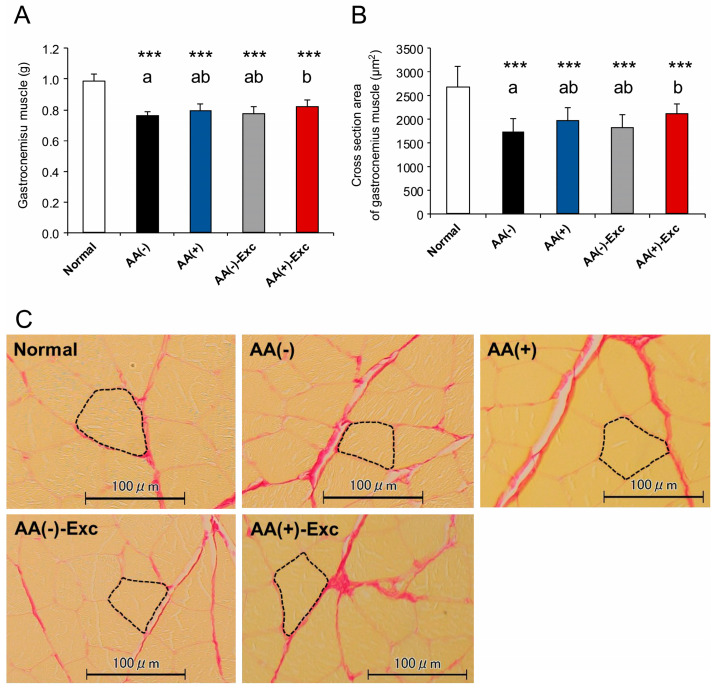
Effects of AA and/or Exc on gastrocnemius muscle mass, myofiber CSA, and histology. (**A**) Gastrocnemius muscle mass. (**B**) Measurement of gastrocnemius muscle CSA. (**C**) Representative micrographs of gastrocnemius muscle, as determined by Sirius red staining. Scale bar = 100 μm. Data are presented as means ± SDs (n = 9–10 per group). *** *p* < 0.001, compared to normal group using Dunnett-type multiple comparison tests among all groups. Different letters indicate significant differences among postoperative sarcopenic rats in AA (−), AA (+), AA (−)-Exc, and AA (+)-Exc groups using Tukey-type multiple comparison tests (*p* < 0.05).

A significant decrease in skeletal muscle mass was observed in the gastrocnemius, soleus, EDL, plantaris, and TA muscles of all postoperative sarcopenic rat model groups compared with the normal group (*p* < 0.05 and *p* < 0.001, respectively). Among the postoperative sarcopenic rat model groups, gastrocnemius and TA muscle masses in the AA (+)-Exc group were significantly higher than those in the AA (−) group (*p* < 0.05). The soleus muscle masses in the AA (−)-Exc and AA (+)-Exc groups were also significantly higher than those in the AA (−) group (*p* < 0.05), whereas there was no difference in the masses of the EDL and plantaris muscles among the postoperative sarcopenic rat model groups (Figure 2A and Table S1).

The myofiber CSA of the gastrocnemius muscle exhibited a change similar to that of muscle mass, indicating a significant decrease in all the postoperative sarcopenic rat model groups compared with the normal group (*p* < 0.001). The CSA of the gastrocnemius muscle in the AA (+)-Exc group was significantly higher than that in the AA (−) group (*p* < 0.05; Figure 2B,C). The number of measured fibers was as follows: Normal: 135.6 ± 23.2, AA (−): 201.6 ± 30.5, AA (+): 178.0 ± 23.3, AA (−)-Exc: 198.6 ± 28.9, and AA (+)-Exc: 168.7 ± 17.7 of fibers (means ± SDs, n = 9–10 per group).

### 3.3. Hindlimb Muscle Strength

**Figure 3:**
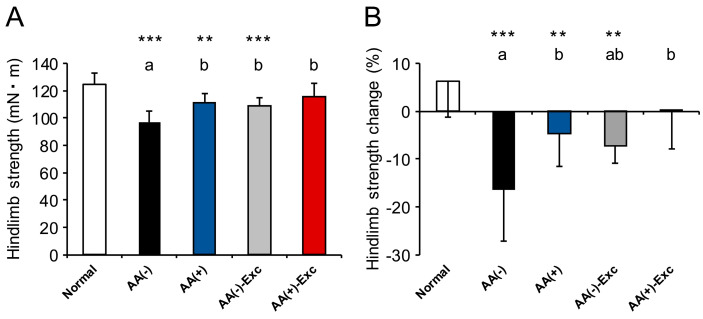
Effects of AA and/or Exc on hindlimb muscle strength. (**A**) Hindlimb muscle strength at 5 days post-treatment (day 5). (**B**) The change in hindlimb strength from before surgery and HS (day −1) to sacrifice (day 5). Data are presented as means ± SDs (n = 9–10 per group). ** *p* < 0.01, *** *p* < 0.001, compared to the normal group using Dunnett-type multiple comparison tests among all groups. Different letters indicate significant differences among the postoperative sarcopenic rats in the AA (−), AA (+), AA (−)-Exc, and AA (+)-Exc groups using Tukey-type multiple comparison tests (*p* < 0.05).

The final hindlimb muscle strength and rates of change in muscle strength in the AA (−), AA (+), and AA (−)-Exc groups were significantly lower than those in the normal group (*p* < 0.01 or *p* < 0.001, respectively). In the AA (+)-Exc group, no significant difference was observed in the final hindlimb muscle strength and rate of change in muscle strength compared with the normal group. A comparison of the postoperative sarcopenic model groups showed that the final hindlimb muscle strength in the AA (−) group was significantly lower than that in the other groups (*p* < 0.05), and the rates of change in muscle strength in the AA (+) and the AA (+)-Exc groups were significantly higher than those in the AA (−) group (*p* < 0.05; Figure 3).

### 3.4. mRNA Expression of Atrogenes

**Figure 4:**
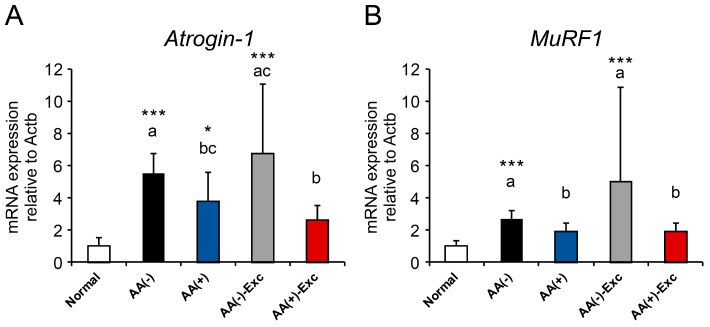
Effects of AA and/or Exc on atrogene mRNA expression levels mRNA expression levels of (**A**) Atrogin-1 and (**B**) MuRF1 in gastrocnemius muscle. Data are presented as means ± SDs (n = 9–10 per group). * *p* < 0.05, *** *p* < 0.001, compared to the normal group using Dunnett-type multiple comparison tests among all groups. Different letters indicate significant differences among the postoperative sarcopenic rats in the AA (−), AA (+), AA (−)-Exc, and AA (+)-Exc groups using Tukey-type multiple comparison tests (*p* < 0.05).

In the gastrocnemius muscle, the mRNA expression of muscle atrophy markers associated with the ubiquitin–proteasome system, such as Atrogin-1 and MuRF1, was examined. The mRNA expression levels of Atrogin-1 and MuRF1 were significantly upregulated in the AA (−) and AA (−)-Exc groups compared with those in the normal group (*p* < 0.001). Notably, the mRNA expression level of Atrogin-1 in the AA (+)-Exc group was significantly lower than that in the AA (−) and AA (−)-Exc groups (*p* < 0.05, Figure 4A). As for the mRNA expression of MuRF1 in comparison to the postoperative sarcopenic model groups, the AA (+) and AA (+)-Exc groups were significantly downregulated compared with the AA (−) group (*p* < 0.05, Figure 4B).

### 3.5. mRNA Expression of Autophagy-Related Genes

**Figure 5:**
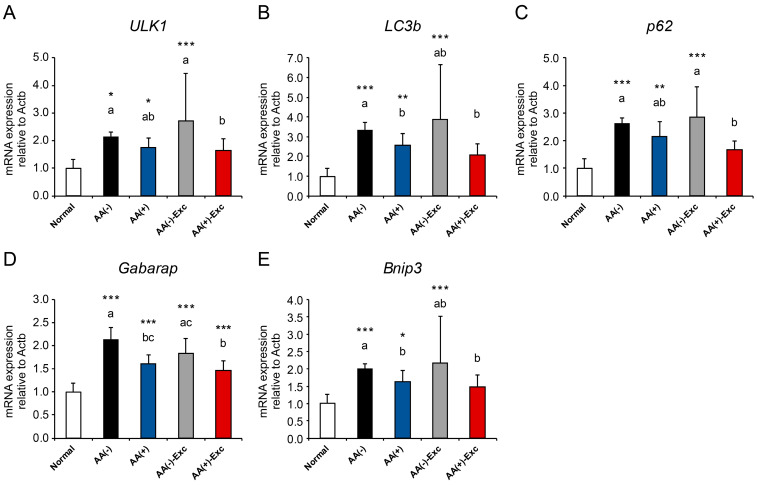
Effects of AA and/or Exc on the mRNA expression levels of autophagy-related genes. mRNA expression levels of (**A**) ULK1, (**B**) LC3b, (**C**) p62, (**D**) Gabarap, and (**E**) Bnip3 in the gastrocnemius muscle. Data are presented as means ± SDs (n = 9–10 per group). * *p* < 0.05, ** *p* < 0.01, *** *p* < 0.001, compared to the normal group using Dunnett-type multiple comparison tests among all groups. Different letters indicate significant differences among the postoperative sarcopenic rats in the AA (−), AA (+), AA (−)-Exc, and AA (+)-Exc groups using Tukey-type multiple comparison tests (*p* < 0.05).

The mRNA expression of muscle atrophy markers associated with autophagy was measured in the gastrocnemius muscle. Except for the AA (+)-Exc group, ULK1, LC3b, p62, and Bnip3 mRNA expression levels were significantly upregulated in the postoperative sarcopenic model groups (*p* < 0.05, *p* < 0.01, and *p* < 0.001, respectively; Figure 5A–C,E). Gabarap mRNA expression was significantly upregulated in all the postoperative sarcopenic model groups (*p* < 0.001, Figure 5D). A comparison of the postoperative sarcopenic model groups revealed that all measured autophagy-related genes were significantly downregulated in the AA (+)-Exc group compared with those in the AA (−) group (*p* < 0.05, Figure 5A–E).

### 3.6. mRNA Expression of FOXO Transcription Factors and Its Target Gene

**Figure 6:**
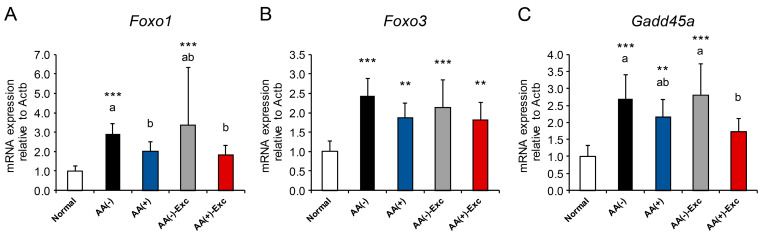
Effects of AA and/or Exc on the mRNA expression of FOXO transcription factors and their target genes. mRNA expression levels of (**A**) Foxo1, (**B**) Foxo3, and (**C**) Gadd45a in the gastrocnemius muscles. Data are presented as means ± SDs (n = 9–10 per group). ** *p* < 0.01, *** *p* < 0.001, compared to the normal group using Dunnett-type multiple comparison tests among all groups. Different letters indicate significant differences among the postoperative sarcopenic rats in the AA (−), AA (+), AA (−)-Exc, and AA (+)-Exc groups using Tukey-type multiple comparison tests (*p* < 0.05).

The mRNA expression levels of the FOXO transcription factors Foxo1 and Foxo3 and its target gene Gadd45a were measured in the gastrocnemius muscle. Foxo1 mRNA was significantly upregulated in the AA (−) and AA (−)-Exc groups compared with the normal group (*p* < 0.001). That is, in the AA (+) and AA (+)-Exc groups, it was significantly downregulated compared with that in the AA (−) group (*p* < 0.05, Figure 6A). Foxo3 mRNA was significantly upregulated in all postoperative sarcopenic model groups compared with the normal group (*p* < 0.01 or *p* < 0.001, respectively); however, no significant difference was observed between the postoperative sarcopenic model groups (Figure 6B). The mRNA expression levels of Gadd45a in the AA (+)-Exc group were not significantly higher than those in the normal group but were significantly lower than those in the AA (−) and AA (−)-Exc groups (*p* < 0.05, Figure 6C).

### 3.7. mRNA Expression of Eif4ebp1 and Phosphorylation of 4E-BP1

**Figure 7:**
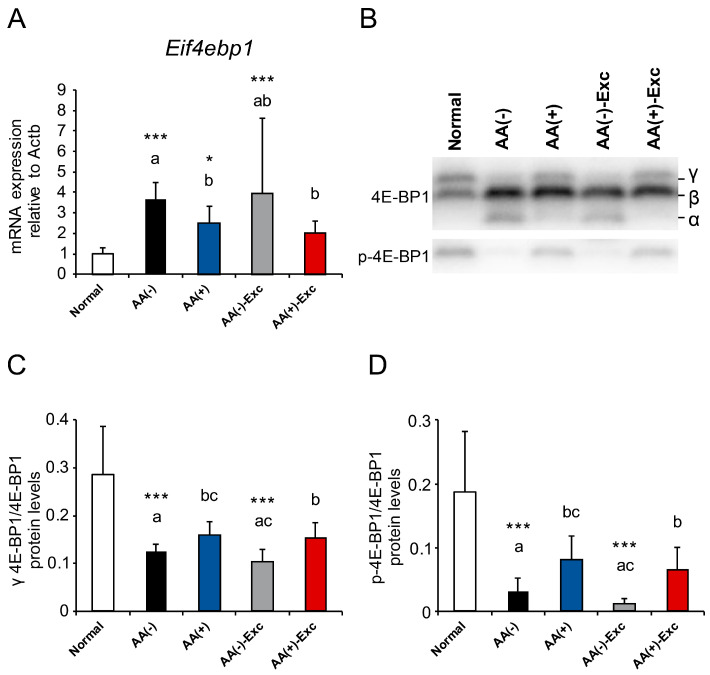
Effects of AA and/or Exc on mRNA expression of Eif4ebp1 and phosphorylation of 4E-BP1. (**A**) mRNA expression levels of Eif4ebp1. (**B**) Representative immunoblot images of 4E-BP1 and p-4E-BP1 expression. Protein levels of phosphorylated 4E-BP1: (**C**) γ forms to total 4E-BP1 protein and (**D**) phospho 4E-BP1^Ser65^ to total 4E-BP1 protein in the gastrocnemius muscle. Data are presented as means ± SDs (n = 9–10 per group). * *p* < 0.05, *** *p* < 0.001, compared to normal group using Dunnett-type multiple comparison tests among all groups. Different letters indicate significant differences among postoperative sarcopenic rats in AA (−), AA (+), AA (−)-Exc, and AA (+)-Exc groups using Tukey-type multiple comparison tests (*p* < 0.05).

In the gastrocnemius muscle, the mRNA expression levels of Eif4ebp1 and phosphorylation levels of 4E-BP1 were measured. This protein is a downstream factor of the mechanistic target of rapamycin complex 1 (mTORC1), which is associated with the initiation of protein synthesis. The mRNA expression levels of Eif4ebp1 were significantly higher in the postoperative sarcopenic model groups, except for AA (+)-Exc, than in the normal group (*p* < 0.05, and *p* < 0.001, respectively). Comparisons among the postoperative sarcopenic model groups showed that the levels in the AA (+) and AA (+)-Exc groups were significantly lower than those in the AA (−) group (*p* < 0.05, Figure 7A). The phosphorylation levels of 4E-BP1 were assessed by comparing highly phosphorylated γ forms in the total 4E-BP1 protein and phospho-4E-BP1 (Ser65) to the total 4E-BP1 protein expression levels (Figure 7B). Both measurements showed the same pattern of results: the phosphorylation levels of 4E-BP1 in the AA (−) and AA (−)-Exc groups were significantly lower than those in the normal group (*p* < 0.001), whereas those in the AA (+)-Exc group were significantly higher than those in the AA (−) and AA (−)-Exc groups (*p* < 0.05, Figure 7C,D).

### 3.8. mRNA Expression of Myosin Heavy Chain Isoforms

**Figure 8:**
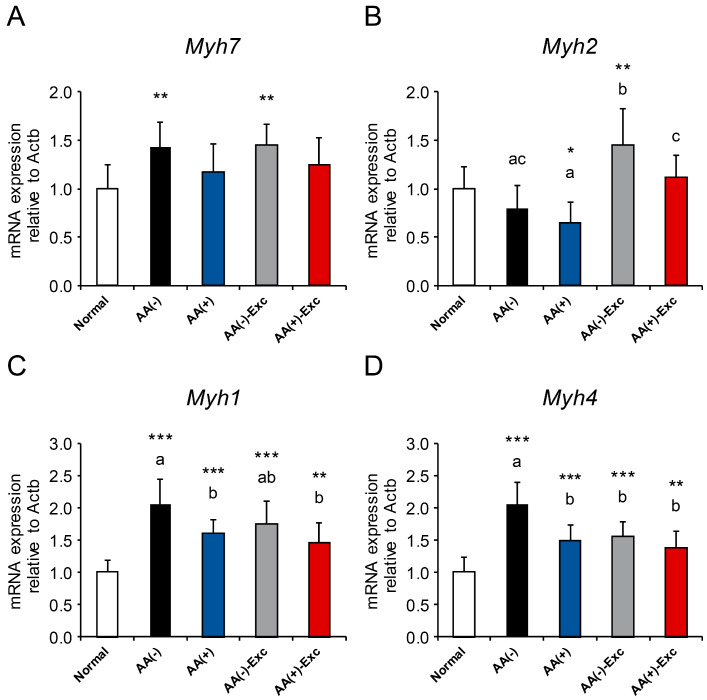
Effects of AA and/or Exc on mRNA expression of myosin heavy chain isoforms. mRNA expression levels of (**A**) Myh7, (**B**) Myh2, (**C**) Myh1, and (**D**) Myh4 in gastrocnemius muscles. Data are presented as means ± SDs (n = 9–10 per group). * *p* < 0.05, ** *p* < 0.01, *** *p* < 0.001, compared to normal group using Dunnett-type multiple comparison tests among all groups. Different letters indicate significant differences among postoperative sarcopenic rats in AA (−), AA (+), AA (−)-Exc, and AA (+)-Exc groups using Tukey-type multiple comparison tests (*p* < 0.05).

The mRNA expression levels of myosin heavy chain isoforms Myh7 (type I fiber), Myh2 (type IIA fiber), Myh1 (type IIX fiber), and Myh4 (type IIB fiber) were measured in the gastrocnemius muscle. Myh1 and Myh4 mRNA levels were significantly upregulated in all the postoperative sarcopenic rat model groups compared with those in the normal group (*p* < 0.01 or *p* < 0.001, respectively; Figure 8C,D). However, among the postoperative sarcopenic rat model groups, the mRNA expression of Myh1 was significantly downregulated in the AA (+) and AA (+)-Exc groups compared with that in the AA (−) group (*p* < 0.05, Figure 8C). Furthermore, a significantly lower mRNA expression level of Myh4 was observed in the AA (+), AA (−)-Exc, and AA (+)-Exc groups than in the AA (−) group (*p* < 0.05, Figure 8D).

### 3.9. Plasma AA Concentrations

Plasma branched-chain amino acid (BCAA) concentrations were significantly lower in the AA (−) and AA (−)-Exc groups than in the normal group (*p* < 0.05 and *p* < 0.001, respectively). Plasma essential amino acid (EAA) concentrations in the AA (−) and AA (−)-Exc groups were also significantly lower than those in the normal group (*p* < 0.05 and *p* < 0.01, respectively). Plasma non-essential amino acid (NEAA) concentrations were significantly higher in the AA (−) group (*p* < 0.01) and significantly lower in the AA (+) and AA (+)-Exc groups than in the normal group (*p* < 0.001). Hence, the administration of AAs in the AA (+) and AA (+)-Exc groups contributed to the maintenance of plasma BCAA and EAA levels similar to those observed in the normal group (Table 2). The individual AA concentrations are listed in Table S2.

## 4. Discussion

The development of sarcopenia following surgery can lead to adverse patient outcomes. Therefore, preventing sarcopenia after surgery is a major concern for hospitalized patients. The rat models of postoperative sarcopenia used in this study appeared to mimic the clinical setting of patients who were on bed rest following surgery. This study demonstrated that AAs comprising PPN or Exc alone partially ameliorated postoperative sarcopenia in the rat model. Moreover, the combination of AAs comprising PPN with Exc was found to be more effective in preventing postoperative sarcopenia. This mechanism could be explained by these factors attenuating the dysregulated mRNA expression of muscle atrophy-related genes, including ubiquitin proteasome, autophagy, translation initiation factor, and myosin heavy chain isoforms.

AAs comprising PPN, which is hypocaloric with sufficient protein intake, improved the rate of change in body weight, lean mass loss, and muscle strength in this study. A previous study, using aged mice as an age-related sarcopenia model, showed that the oral supplementation of AAs such as BCAAs plus alanine or a dipeptide of alanine improved muscle atrophy [33]. Hence, the administration of AAs could be effective for primary and secondary sarcopenia in view of these animal studies. Furthermore, the combination of parenteral AA administration with Exc had additive effects on improving body weight, lean mass, gastrocnemius muscle mass, gastrocnemius myofiber CSA, and muscle strength compared with the absence of AAs and Exc. It is also important to note that the combined effect was demonstrated under hypocaloric energy intake in a short postoperative period using a PPN solution. Assessments of muscle strength and mass are important diagnostic criteria for sarcopenia [2,34]. Importantly, in the present study, parenteral AA infusion combined with Exc improved not only muscle mass but also muscle strength, which is principally related to the adverse outcomes of sarcopenia [2], in a postoperative sarcopenic rat model.

The ubiquitin–proteasome and autophagy–lysosome systems play key roles in protein degradation during muscle atrophy [14,15,16]. The upregulation of ubiquitin ligases and autophagy-related mRNA or protein expression was observed in animal models of muscle disuse and human bed rest conditions [15,29,35,36,37,38]. In this study, we measured mRNA expressions of atrogenes (Atrogin-1, MuRF1), autophagy-related genes (ULK1, LC3B, p62, Gabarap, and Bnip3), and FOXO transcription factors (Foxo1, Foxo3, and Gadd45a) which control both systems [17,39,40]. We demonstrated the upregulation of the mRNA expression levels of atrogenes and autophagy-related genes in the gastrocnemius muscle of postoperative sarcopenic rat models, especially in the AA (−) group. The upregulation of MuRF1 mRNA expression has been reported in old, malnourished rats [41]. Additionally, autophagy is also induced by nutritional stress, especially in AA deficiency in the liver [42] and skeletal muscle of low-protein diet- and single essential AA-deprived diet-fed animals [43,44]. Our results indicate that AAs alone partially and in combination with Exc suppressed muscle atrophy-related markers; however, Exc alone failed to suppress this effect. These results may contribute to the development of interventions aimed at the suppression of muscle atrophy and weakness induced by postoperative sarcopenia. Therefore, not only Exc but appropriate nutritional management, such as AA infusion and Exc, seems to be an effective treatment strategy for attenuating the ubiquitin–proteasome and autophagy–lysosome systems. The elevation of Foxo1 and Gadd45a mRNA levels was also suppressed by the combination of parenteral AA and Exc; however, Foxo3 mRNA levels were not altered by parenteral AA alone, exercise alone, or a combination of these. This difference may be because the timing of the changes differs in Foxo1 and Foxo3 mRNA expression after HS [45].

This study demonstrated an increase in Eif4ebp1 mRNA expression and a decrease in the phosphorylation levels of 4E-BP1, indicating a negative effect on protein synthesis in the postoperative sarcopenic rat model without AAs, regardless of the presence or absence of Exc. Previous studies have also shown the elevated mRNA expression of Eif4ebp1 [46] and attenuation of the phosphorylation level of 4E-BP1 [47,48] in the skeletal muscle of HS rats. AA administration alone and with Exc significantly improved the mRNA expression of Eif4ebp1 and phosphorylation levels of 4E-BP1 compared with the AA (−) group, suggesting that AAs contribute to the promotion of translation initiation. However, there were no significant changes in the phosphorylation levels of other protein synthesis-related factors such as Akt and p70S6K in the present study. This could be due to the continuous infusion of the PPN solution and plasma levels of AAs, especially leucine, not reaching the required level for signal transduction.

Additionally, we observed that parenteral AA infusion partially prevented the aberrant mRNA expression of myosin heavy chain isoforms observed in the gastrocnemius muscles of the postoperative sarcopenic rat models. It has been reported that muscle disuse induces a shift in slow-to-fast changes in muscle fibers [19]. In the present study, the expression levels of Myh1 (type IIX fiber) and Myh4 (fast-twitch type IIB fiber) were upregulated, indicating a shift to a fast transition of muscle fibers. Parenteral AA infusion with or without Exc modified the mRNA expression levels of Myh1 and Myh4 compared with the AA (−) group. Theilen et al. [49] observed a significant increase in the mRNA expression of Myh4 and the attenuation of this increase by Exc prior to HS, in the soleus muscle of HS mice. Another report by Vilchinskaya et al. [50] also indicated that the upregulation of Myh1 and Myh4 was induced by HS, and the inhibition of Foxo1 attenuated this upregulation in the skeletal muscles of rats. In our experiment, Foxo1 mRNA levels were suppressed by parenteral AA infusion, which may be related to the prevention of the fast transition of the muscle fiber type through FOXO inhibition in muscle atrophy.

Plasma AA analysis demonstrated that the parenteral AA-administered groups maintained their plasma BCAA and EAA levels, which were similar to those of the normal group. In contrast, the postoperative sarcopenic groups without AA administration exhibited a decrease in BCAA and EAA levels, accompanied by an increase in NEAA levels. A reduction in EAA levels has been previously reported in severely frail elderly patients [51]. The increase in NEAA levels can be attributed to the elevation of plasma glutamine (Table S2), which is a highly enriched AA in skeletal muscles. Elevated plasma glutamine levels have also been reported in elderly individuals with sarcopenia [52] and in severely frail elderly patients [51].

The strength of this study is that we focused on postoperative sarcopenia and used animal models in the first place. Our preliminary study demonstrated that combining the surgical procedure with HS led to a severe decrease in muscle strength or muscle mass compared to surgery or HS alone. Furthermore, we were able to evaluate the effect of AAs comprising PPN and/or Exc and explore their underlying mechanisms. On the other hand, this study had some limitations. First, we could not perform a comparison of the effects of parenteral AA infusion and/or Exc on other skeletal muscles, except for the gastrocnemius, as more improvement using AAs combined with Exc was seen in the gastrocnemius muscle than in the other muscles. Hence, further investigations are needed to determine the effects of parenteral AAs and/or Exc on other skeletal muscle tissues in this model. In addition, the gastrocnemius muscle is composed of a mixture of fiber types, including types I, IIa, IIx, and IIb, although it predominantly contains fast-twitch-type fibers [53]. The results of the present study, which focused on the gastrocnemius muscle sample, particularly in terms of the mRNA expression of myosin heavy chain isoforms, suggest that this may reflect the presence of fast-twitch-type fibers. However, further evaluation is needed to confirm the fiber type-specific effect of AA and/or Exc. Second, the intermittent loading of Exc was conducted according to the methods proposed in previous studies [29,30]; however, it was difficult to obtain a unified Exc volume in individual animals. Hence, the combined effects of Exc itself may be altered depending on the individual and study. However, the combination of AAs and Exc was more effective than other treatments in the present study.

## 5. Conclusions

Our study demonstrated that the postoperative sarcopenic rat models exhibited a marked reduction in both muscle strength and mass, particularly following the administration of an AA-free solution on its own. In contrast, even during a brief postoperative period, the parenteral administration of AAs with Exc, not Exc alone, seemed to be effective in preventing postoperative sarcopenia.

Further studies using older animals are necessary to establish a more accurate understanding of postoperative sarcopenia in elderly patients in a clinical setting. Additionally, it is necessary to examine not only the dosage of AA administration but also the impact of PN alone as well as PN combined with EN. Future clinical trials are warranted to validate our non-clinical study.

## Figures and Tables

**Table 1:** Composition of experimental solution (/1000 mL) and daily dosage.

	AA (−)	AA (+)	AA (−)-Exc	AA (+)-Exc
**Composition**				
Glucose (%, *w*/*v*)	10.0	7.5	10.0	7.5
Amino acids (%, *w*/*v*)	0	3.0	0	3.0
Total calories (kcal)	400	420	400	420
**Daily Dosage**				
Volume (mL/kg/d)	245	233.3	245	233.3
Total energy (kcal/kg/d)	98	98	98	98
Glucose (g/kg/d)	24.5	17.5	24.5	17.5
Amino acids (g/kg/d)	0	7.0	0	7.0

Commercially available 10% glucose and electrolytes solution (AA (−)) or 7.5% glucose, 3% amino acids, and electrolytes solution (AA (+)) were used. The daily dosage of energy 98 kcal/kg/d was the same in AA (−), AA (+), AA (−)-Exc, and AA (+)-Exc groups.

**Table 2:** Plasma amino acid concentrations (nmol/mL).

	Normal	AA (−)	AA (+)	AA (−)-Exc	AA (+)-Exc
BCAA	524.7 ± 104.9	338.6 ± 25.5 **^, a^	525.7 ± 39.7 ^b^	318.9 ± 32.5 ***^, a^	489.4 ± 37.8 ^b^
EAA	1608.1 ± 193.2	1364.1 ± 71.4 *^, a^	1757.2 ± 91.0 ^b^	1311.7 ± 83.8 **^, a^	1683.3 ± 88.3 ^b^
NEAA	2580.1 ± 211.5	2826.2 ± 174.3 **^, a^	2205.6 ± 98.5 ***^, b^	2620.2 ± 158.9 ^c^	2097.0 ± 108.9 ***^, b^

BCAA: branched-chain amino acid (valine, leucine, and isoleucine); EAA: essential amino acid (isoleucine, threonine, tryptophan, valine, histidine, phenylalanine, methionine, lysine, and leucine); NEAA: non-essential amino acid (asparagine, aspartic acid, alanine, arginine, glycine, glutamine, glutamic acid, cysteine, serine, tyrosine, and proline). Data are means ± SDs (n = 9–10 per group). * *p* < 0.05, ** *p* < 0.01, *** *p* < 0.001, compared to the normal group by Dunnett-type multiple comparison tests among all groups. Different letters indicate significant differences among the postoperative sarcopenic rats in AA (−), AA (+), AA (−)-Exc, and AA (+)-Exc groups by Tukey-type multiple comparison tests (*p* < 0.05).

## Data Availability

The data presented in this study are available on request from the corresponding author. The data are not publicly available due to privacy.

## Supplementary Materials

The following supporting information can be downloaded at https://www.mdpi.com/article/10.3390/nu16081218/s1, Table S1: Hindlimb skeletal muscle mass; Table S2: Individual plasma amino acid concentrations (nmol/mL).
