# Supplementary material for: Combination of Parenteral Amino Acid Infusion and Intermittent Loading Exercise Ameliorates Progression of Postoperative Sarcopenia in Rat Model
Source: Nutrients. 2024 Apr 19;16(8):1218. doi: 10.3390/nu16081218 (PMC11054099; doi:10.3390/nu16081218)
Supplement: Supplementary file 1 [file nutrients-16-01218-s001.zip › nutrients-2905114-supplementary.pdf]

Supplementary Material

Supplementary Table S1. Hindlimb skeletal muscle mass.

	Normal	AA (−)	AA (+)	AA (−)-Exc	AA (+)-Exc
Soleus muscle mass (g)	0.081 ± 0.008	0.057 ± 0.005 ***, a	0.060 ± 0.006 ***, ab	0.065 ± 0.005 ***, b	0.066 ± 0.004 ***, b
EDL muscle mass (g)	0.091 ± 0.005	0.080 ± 0.007 ***	0.085 ± 0.004 *	0.085 ± 0.004 *	0.084 ± 0.004 *
Plantaris muscle mass (g)	0.191 ± 0.011	0.149 ± 0.005 ***	0.155 ± 0.009 ***	0.155 ± 0.011 ***	0.159 ± 0.011 ***
TA muscle mass (g)	0.344 ± 0.021	0.288 ± 0.010 ***, a	0.302 ± 0.018 ***, ab	0.295 ± 0.018 ***, ab	0.310 ± 0.019 ***, b

EDL: extensor digitorum longus; TA: tibialis anterior.

Data are means ± SDs (n=9-10 per group). * p <0.05, *** p <0.001, compared to Normal group by Dunnett type multiple comparison tests among all groups. Different letters indicate significant differences among the postoperative sarcopenic rats in AA (−), AA (+), AA (−)-Exc, and AA (+)-Exc groups by Tukey type multiple comparison tests (p <0.05).

Supplementary Table S2. Individual plasma amino acid concentrations (nmol/mL).

	Normal	AA (−)		AA (+)		AA (−)-Exc		AA (+)-Exc	
Isoleucine	105.9 ± 21.1	67.9 ± 7.0	***, a	100.1 ± 7.6	b	64.4 ± 7.4	***, a	95.1 ± 8.2	b
Threonine	281.3 ± 41.7	267.7 ± 24.4	a	336.5 ± 26.8	***, b	264.6 ± 19.3	a	337.8 ± 32.1	***, b
Tryptophan	96.6 ± 10.0	81.5 ± 7.1	***, a	108.6 ± 3.8	**, b	78.9 ± 5.2	***, a	110.7 ± 7.1	***, b
Valine	252.5 ± 58.7	148.9 ± 11.1	***, a	239.5 ± 18.3	b	140.5 ± 14.0	***, a	223.2 ± 16.2	b
Histidine	79.4 ± 8.6	81.6 ± 4.2	a	83.4 ± 5.2	a	72.2 ± 3.9	b	78.6 ± 3.3	a
Phenylalanine	55.1 ± 2.9	60.3 ± 2.7	ac	72.0 ± 4.3	***, b	59.2 ± 10.0	a	68.5 ± 3.2	***, bc
Methionine	43.9 ± 3.9	50.5 ± 4.4	a	62.7 ± 3.3	***, b	50.0 ± 8.4	a	58.9 ± 2.7	***, ab
Lysine	527.2 ± 38.5	483.9 ± 31.5	*, a	568.4 ± 33.8	*, b	468.0 ± 34.9	**, a	539.5 ± 31.8	b
Leucine	166.2 ± 25.4	121.9 ± 8.3	*, a	186.1 ± 15.5	b	114.0 ± 11.6	**, a	171.1 ± 14.4	b
Asparagine	80.1 ± 12.4	62.9 ± 5.8	a	38.6 ± 2.6	***, b	57.6 ± 4.7	c	37.1 ± 2.9	***, b
Aspartic acid	11.9 ± 4.8	11.0 ± 1.2	a	7.1 ± 0.7	**, b	11.1 ± 1.8	a	7.2 ± 0.6	**, b
Alanine	762.5 ± 118.2	714.3 ± 65.7	a	545.8 ± 48.9	***, b	629.7 ± 74.0	c	493.1 ± 44.8	***, b
Arginine	131.6 ± 14.1	157.2 ± 17.5	*, a	242.8 ± 24.3	***, b	145.0 ± 11.6	a	227.9 ± 18.7	***, b
Glycine	153.3 ± 24.5	234.3 ± 24.2	***	220.6 ± 10.8	***	235.5 ± 27.3	***	217.2 ± 17.2	***
Glutamine	632.1 ± 40.0	870.0 ± 64.2	*, a	520.7 ± 25.3	b	808.7 ± 80.6	a	511.1 ± 35.3	*, b
Glutamic acid	82.3 ± 11.1	83.8 ± 9.5	a	59.9 ± 3.9	***, b	83.6 ± 7.9	a	62.0 ± 6.5	***, b
Cystine	32.5 ± 4.1	30.8 ± 4.4		35.1 ± 3.3		31.9 ± 5.8		33.1 ± 4.0	
Serine	297.4 ± 24.3	422.5 ± 32.3	**, a	290.3 ± 14.1	b	391.7 ± 43.3	*, a	282.2 ± 19.7	b
Tyrosine	112.3 ± 23.5	89.6 ± 10.8	a	80.9 ± 7.2	**, ab	79.8 ± 10.3	**, ab	70.7 ± 6.6	***, b
Proline	284.1 ± 97.3	149.9 ± 12.7	**, a	163.9 ± 9.7	b	145.6 ± 10.9	***, a	155.6 ± 9.8	*, ab

Data are means ± SDs (n=9-10 per group). * p <0.05, ** p <0.01, *** p <0.001, compared to Normal group by Dunnett type multiple comparison tests among all groups. Different letters indicate significant differences among the postoperative sarcopenic rats in AA (−), AA (+), AA (−)-Exc, and AA (+)-Exc groups by Tukey type multiple comparison tests (p <0.05).
